# Suppression of EGFR/PKC-δ/NF-κB Signaling Associated With Imipramine-Inhibited Progression of Non-Small Cell Lung Cancer

**DOI:** 10.3389/fonc.2021.735183

**Published:** 2021-10-26

**Authors:** Po-Fu Yueh, Yuan-Hao Lee, I-Tsang Chiang, Wei-Ting Chen, Keng-Li Lan, Cheng-Hsien Chen, Fei-Ting Hsu

**Affiliations:** ^1^ Institute of Traditional Medicine, School of Medicine, National Yang-Ming Chiao Tung University, Taipei, Taiwan; ^2^ Department of Radiation Physics, Division of Radiation Oncology, The University of Texas MD Anderson Cancer Center, Houston, TX, United States; ^3^ Department of Radiation Oncology, Show Chwan Memorial Hospital, Changhua, Taiwan; ^4^ Department of Radiation Oncology, Chang Bing Show Chwan Memorial Hospital, Changhua, Taiwan; ^5^ Department of Medical Imaging and Radiological Sciences, Central Taiwan University of Science and Technology, Taichung, Taiwan; ^6^ Department of Psychiatry, Zuoying Branch of Kaohsiung Armed Forces General Hospital, Kaohsiung, Taiwan; ^7^ Department of Oncology, Taipei Veterans General Hospital, Taipei, Taiwan; ^8^ Surgical Department of Show Chwan Memorial Hospital, Changhua, Taiwan; ^9^ Department of Biological Science and Technology, China Medical University, Taichung, Taiwan

**Keywords:** imipramine, non-small cell lung cancer (NSCLC), EGFR - epidermal growth factor receptor, PKC-δ-Protein kinase C delta, NF-κB – nuclear factor kappa B

## Abstract

**Background:**

Anti-depressants have been reported to own anti-tumor potential types of cancers; however, the role of imipramine in non-small cell lung cancer (NSCLC) has not been elucidated. Epidermal growth factor receptor (EGFR) was known to be one of the key regulators that control NSCLC progression. Whether EGFR would be the target of imipramine for suppressing tumor signaling transduction and results in anti-tumor potential is remaining unclear.

**Methods:**

We used CL-1-5-F4 cells and animal models to identify the underlying mechanism and therapeutic efficacy of imipramine. Cytotoxicity, apoptosis, invasion/migration, DNA damage, nuclear translocation of NF-κB, activation of NF-κB, phosphorylation of EGFR/PKC-δ/NF-κB was assayed by MTT, flow cytometry, transwell, wound healing assay, comet assay, immunofluorescence staining, NF-κB reporter gene assay and Western blotting, respectively. Tumor growth was validated by CL-1-5-F4/*NF-κB-luc2* bearing animal model.

**Results:**

Imipramine effectively induces apoptosis of NSCLC cells *via* both intrinsic and extrinsic apoptosis signaling. DNA damage was increased, while, invasion and migration potential of NSCLC cells was suppressed by imipramine. The phosphorylation of EGFR/PKC-δ/NF-κB and their downstream proteins were all decreased by imipramine. Similar tumor growth inhibition was found in imipramine with standard therapy erlotinib (EGFR inhibitor). Non-obvious body weight loss and liver pathology change were found in imipramine treatment mice.

**Conclusion:**

Imipramine-triggered anti-NSCLC effects in both *in vitro* and *in vivo* model are at least partially attributed to its suppression of EGFR/PKC-δ/NF-κB pathway.

**Graphical Abstract:**
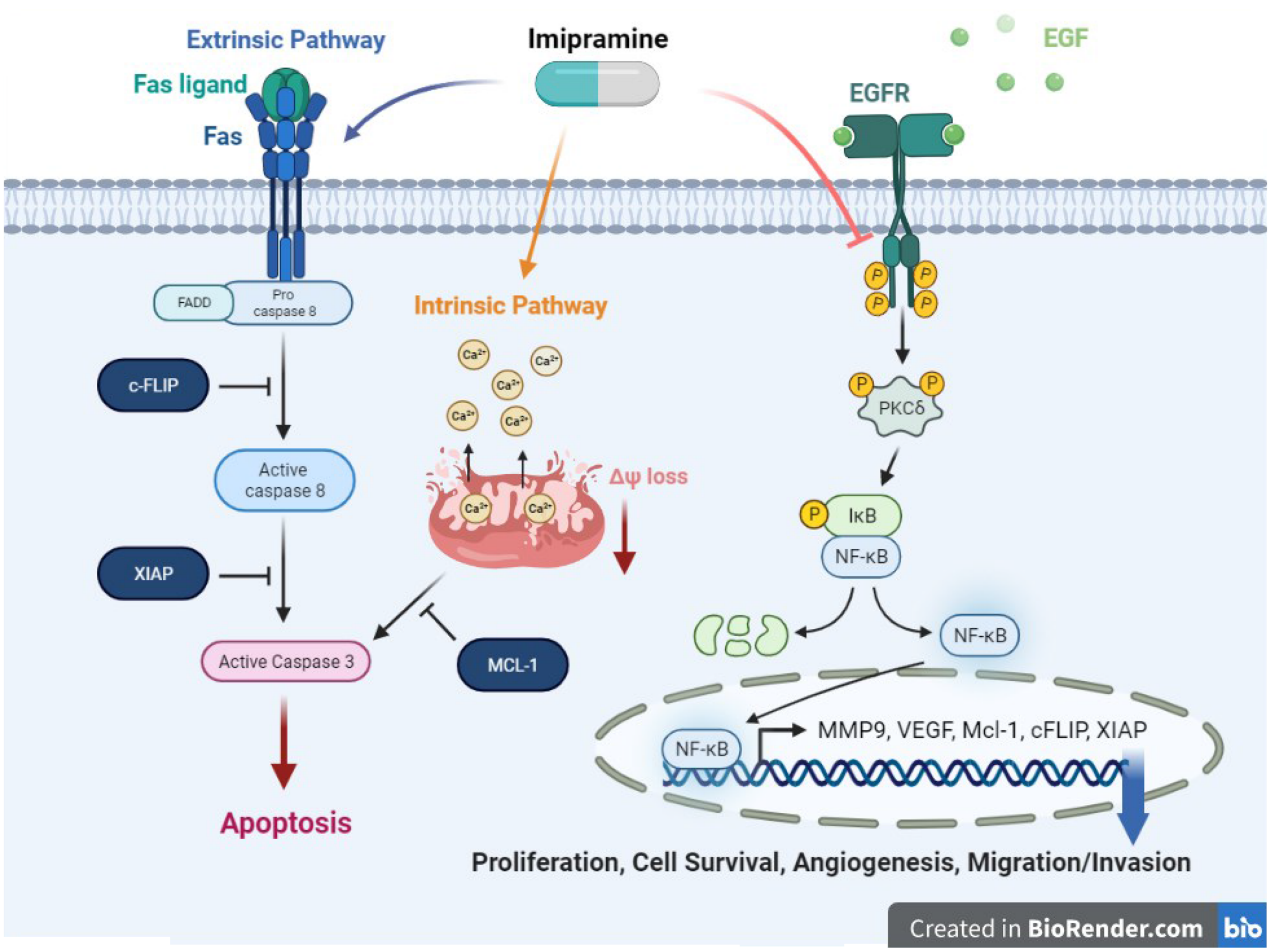
Imipramine, an anti-depressant, could induce extrinsic and intrinsic apoptosis pathway in NSCLC. Moreover, imipramine has potential to inhibit tumor progression including proliferation, cell survival, angiogenesis, and migration/invasion, through suppress EGFR/PKC-δ/NF-κB pathway.

## Background

Antidepressant medications in major classes of monoamine oxidase inhibitors (MAOIs), tricyclic antidepressants (TCAs), selective serotonin reuptake inhibitors (SSRIs), serotonin-norepinephrine reuptake inhibitors (SNRIs) and atypical antidepressants are used to improve symptoms of depression by blocking breakdown or reuptake of neurotransmitters (1–3). In addition to the pharmacological effects on neurobiological systems, the roles of antidepressant medications played in oncology have also been extensively investigated (4, 5).

Indicated by cell and animal models, antidepressants as potential anti-cancer agents inhibit the growth of hepatocellular carcinoma, glioblastoma, and colorectal cancer through induction of apoptosis, inactivation of oncogenic kinases, and enhancement of anti-tumor immunity (6–8). Fluoxetine, an SSRI, can induce apoptosis of HCC cells through an intrinsic pathway (7). Imipramine, a TCA, can inhibit the progression of glioblastoma cells *via* potentiating dephosphorylation of extracellular regulated protein kinase (ERK) and apoptosis signaling (8). Moreover, increased expression of anticancer cytokines interleukin-12 (IL-12) and interferon-gamma (INFγ) was found to be associated with mirtazapine, an atypical antidepressant that inhibited the growth of CRC cells (6).

Lung cancers, classified into small cell lung cancer (SCLC) and non-small cell lung cancer (NSCLC), are the most common malignancy worldwide (9). Treatment options for lung cancers include surgery, chemotherapy, radiotherapy as well as monoclonal antibodies targeting epidermal growth factor receptor (EGFR), anaplastic lymphoma kinase (ALK), programmed cell death 1 (PD-1) and programmed cell death ligand 1 (PD-L1) (10, 11). TCAs have been shown as adjunct therapeutics to prolong the survival of lung cancer patients receiving chemotherapy (12). Among various TCAs, imipramine was found to mediate SCLC cell death through inhibition of autocrine survival signals including neurotransmitters and their G protein-coupled receptors (13). However, anti-NSCLC efficacy and mechanisms of imipramine remain to be elucidated. Epidermal growth factor receptor (EGFR), a receptor tyrosine kinase, can be activated by its ligands such as EGF, transforming growth factor-α (TGF-α), and amphiregulin. Hyperactive EGFR signaling promotes tumor cell growth, survival, angiogenesis, and metastasis through upregulating activation of downstream kinase cascades (14, 15). It has been shown that suppression of epidermal growth factor receptor (EGFR) signaling in NSCLC correlates with blockade of tumor progression (16). Therefore, the major purpose of this study is to verify whether inhibition of EGFR signaling associates with imipramine-inhibited progression of NSCLC cells.

## Materials and Methods

### Chemicals, Antibodies, and Reagents

Imipramine, MTT (3-(4,5-Dimethylthiazol-2-yl)-2,5-Diphenyltetrazolium Bromide), RNase, dimethyl sulfoxide (DMSO), and Hygromycin B were obtained from Sigma Chemical Co. (St. Louis, MO, USA). Dulbecco’s Modified Eagle Medium/Nutrient Mixture F-12 (DMEM/F-12), fetal bovine serum (FBS), penicillin-streptomycin were purchased from GIBCO^®^/Invitrogen Life Technologies (Carlsbad, CA, USA). Primary antibodies against Matrix metalloproteinase-9 (MMP-9) (AB19016, Millipore), vascular endothelial growth factor (VEGF) (ab1316, Abcam, Cambridge, UK), EGFR (Try 1068) (#2234, Cell signaling, Danvers, MA, USA), EGFR (E-AB-63555, Elabscience, Houston, TX, USA), PKC-δ (Thr507) (E-AB-20968, Elabscience), PKC-δ (E-AB-14675, Elabscience), NF-κB p65 (Ser536) (E-AB-70335, Elabscience), NF-κB p65 (E-AB-22066, Elabscience), cell leukemia-1 (MCL-1) (BV-438, BioVision), cellular FLICE (FADD-like IL-1β-converting enzyme)-inhibitory protein (cFLIP) (D16A8, Cell signaling), X-linked inhibitor of apoptosis protein (XIAP) (PA5-29253, Thermo Fisher Scientific), Fas (E-AB-40063, Elabscience), Fas ligand (FasL) (E-AB-31410, Elabscience), cleaved caspase-3 (E-AB-30004, Elabscience), cleaved caspase-8 (E-AB-22107, Elabscience), cleaved caspase-9 (#9505, Cell Signaling Technology), PARP-1 (#9532, Cell Signaling Technology) and β-actin (sc-47778, Santa Cruz Biotechnology, Dallas, Texas, Waltam, MA, USA) for Western blotting were purchased from different companies as listed. Secondary antibodies, peroxidase affiniPure Goat Anti-Mouse IgG and Goat Anti-Rabbit IgG were purchased from Jackson Immunoresearch Laboratories Inc. (West Grove, PA, USA). D-luciferin was obtained from Caliper Life Science (Hopkinton, MA, USA).

### Cell Culture

CL1-5-F4 cells, non-small cells lung cancer (NSCLC), was kindly provided by Dr. Chia-Lin Hsieh (Taipei Medical University, Taiwan), A549, and NCI-460 provided by Professor Jing-Gung Chung (China Medical University, Taiwan) were used for this present study. Cells were maintained in DMEM/F-12 and F12-K containing 10% FBS, 1% PS and incubated at 37°C in a 95% air and 5% CO_2_ humidified atmosphere.

### 3-(4,5-Dimethylthiazol-2-yl)-2,5-Diphenyltetrazolium Bromide Assay

Cell viability was performed by MTT assay. Cells were seeded into 96-well plates at a density of 1.5x10^4^ cells/well and incubated overnight. Then treated with different concentrations of imipramine for 24 and 48 hr. After treatment, the medium was replaced with 0.5 mg/ml of MTT solution and maintained in the incubator for another four hr. Before the ELISA reader (Thermo Fisher Scientific, Fremont, CA, USA) analysis, the MTT solution was replaced by 100 μl DMSO. The absorbance wavelength of MTT is 570 nm, the blank value was defined as zero (+/–0.1).

### Plasmid Transfection and Stable Clone Selection

CL1-5-F4 cells were transfected with pNF-κB-luc2 (Promega, Madison, WI, USA) vector using JetPEI™ transfection agent (Illkirch, Bas-Rhin, France). One μg pNF-κB-luc2 plasmid dissolved in 150 mM NaCl and mixed with JetPEI™ agent, then incubated for 30 min at 25°C. The DNA mixture was then added to the CL1-5-F4 cells in a 24 well plate dish and incubated for another 24 hr. After transfection, cells were cultured in a medium containing 200 µg/ml of hygromycin B for two weeks. The surviving clones were subsequently seeded into 96-well plates and imaged by IVIS Lumina LT Series (PerkinElmer, Boston, MA, USA). CL1-5-F4 cells with stable NF-κB expression were renamed as CL1-5-F4/*NF-κB-luc2* cells (17).

### NF-ĸB Reporter Gene Assay

CL1-5-F4/*NF-κB-luc2* cells were plated into 96-well (1.5×10^4^/well) overnight and treated with various concentrations of imipramine (0, 50, 100, 150 and 200 μM), erlotinib (10 μM), hEGF (30 ng/ml), rottlerin (20 nM) and Phorbol myristate acetate (PMA) (75 nM) alone or combined with 100 and 150 μM imipramine for 48 hr. After treatment, 100 μl D-luciferin (Promega, Madison, WI, USA) solution (500 μM D-luciferin) was added into each well and incubated for 5 min in the dark at room temperature before image acquisition. NF-κB activation signal was collected for 3 min by IVIS Lumina LT Series and normalized with cell viability.

### Flow Cytometry

CL1-5-F4 cells were seeded into 6-well plates (2×10^5^/well) overnight and treated with 0, 100, and 150 μM of imipramine for 48 hr. Cells were harvested and stained by Annexin V-FITC Apoptosis Detection Kit (Vazyme Biotech Co. Lt, Nanjing, China), DiOC_6_ dye (4 μmol/L), FITC-DEVD-FMK (cleaved caspase-3), Red-IETD-FMK (cleaved caspase-8), FITC-VAD-FMK (cleaved caspase-9), ROS peroxide-sensitive fluorescent probe 2′, 7′-dichlorofluorescein diacetate (DCFH-DA 500 μl at 10 μM, Molecular Probes), and fluo-3-acetomethoxyester (Fluo-3/AM, 2.5 μg/mL) for apoptosis detection, respectively. For cell cycle analysis, cells were fixed by 75% ethanol overnight and followed with 40 μg/ml Propidium iodide (6) stained (contained with 100 μg/ml RNase and 1% Triton X-100). For cleaved PARP-1, cells were fixed by 4% paraformaldehyde and permeabilized by ice-cold 100% methanol before PARP-1 stained (3). After staining, cells were resuspended with 300 μl PBS and the signal intensity of each marker was detected by flow cytometry (BD Biosciences, FACS Calibur, San Jose, CA, USA). Quantification results of fluorescence intensity were measured by FlowJo software (version 7.6.1; FlowJo LLC, Ashland, OR, USA).

### Immunofluorescence Nuclear Translocation Assay

CL1-5-F4 cells were seeded on coverslips overnight and treated with different concentrations of imipramine. Then, the coverslips were fixed with 3.7% paraformaldehyde for 15 min. Then, slices were permeabilized by 0.1% Triton-X100 for 15 min. Block the coverslips with a blocking buffer (Axel Biotechnology Inc.) at 25°C for one hour. The coverslips were then incubated with primary antibodies (1:250 anti-NF-κB) at room temperature for 1 hr. The coverslips were washed by PBS-T (PBS add 0.1% Tween 20) once and PBS twice. Coverslips were incubated with FITC-labeled secondary antibody (Jackson Immunoresearch Laboratories Inc.) at 25°C and protected from light for 1 hr. Finally, slides were fixed with DAPI mounting buffer and allowed it dry in dark. The fluorescence signal from NF-κB in cells were observed and photographed by Zeiss Axio Scope A1 fluorescence microscope (18).

### Trans-Well Migration and Invasion Assay

The 8 μm pore trans-wells were purchased from BD Biosciences (Franklin Lakes, NJ, USA). CL1-5-F4 cells were treated with different concentrations of imipramine for 48 hr and then cells were collected into the upper channel of trans-wells at the number of 5×10^5^, allow 24 hr migration period of CL1-5-F4 cells. Alternatively, to test invasion ability, the upper channel of the trans-well was pre-coated with matrigel. Transwell membranes were then fixed (methanol and acetic acid 3:1) and stained by 0.5% crystal violet. The light microscope (Nikon ECLIPSE Ti-U) was used to photograph the migration and invasion cells at ×100 on the trans-well membrane. The number of migration and invasion cells was calculated using Image J software version 1.50 (National Institutes of Health, Bethesda, MD, USA) (19).

### Wound Healing Assay

Cells were pre-treated imipramine for 48 hr, then seeded the pre-treated cells into 6 well with ibidi culture-inserts (cat: 80241, ibidi GmbH, Gräfelfing, Germany) insert overnight. The 2-well insert was then removed and a cell migration pattern was observed by microscope at 0, 6, 12 and 24 hr. The migration gap area was quantified by Image-J.

### Western Blotting Assay

One million CL1-5-F4 cells were seeded into 10 cm dishes overnight and treated with different concentrations (0, 100 and 150 μM) of imipramine, erlotinib (10 uM) or hEGF (100 ng/ml), respectively, for 48 hr. After treatment, the protein was extracted from each group by NP40 lysis buffer. Total protein was measured using a Pierce BCA Protein Assay Kit (Thermo Fisher Scientific). Proteins were separated by 8-15% SDS-PAGE, transferred on polyvinylidene difluoride (PVDF) membrane (FluoroTrans^®^ Pall Corporation, Port Washington, NY, USA) by the electroblotting system and blocked with 5% fetal calf serum (FCS) in Tris-buffered saline (TBS) containing 0.05% Tween-20. Then, the PVDF membrane was stained by primary antibodies overnight at 4˚C and secondary antibodies for 1 hr at 25°C. Membranes were probed with Immobilon Western Chemiluminescent HRP Substrate (Pierce, Rockford, IL, USA). The chemiluminescence signal from each sample was then detected by the UVP ChemiDoc-It™ (Analytik Jena, Jena, Germany) and their specific band intensities were quantified by VisionWorks (AnalytikJena). Quantification data were all normalized by β-actin expression and averaged by three repeated experiments.

### Establishment of Tumor-Bearing Animal Model

Six-week-old nude mice (N=15) were brought from the National Laboratory Animal Center, Taipei, Taiwan. One million CL1-5-F4/*NF-κB-luc2* cells was suspended 100 μl PBS and subcutaneously inoculated in the right legs of nude mice. When average tumor volume reached about 130 mm^3^, mice were randomly separated into three groups (n=5/group), vehicle group [treated with 0.1% dimethyl sulfoxide (DMSO) by gavage daily for 14 days] and erlotinib group (treated with 20 mg/kg/day by gavage for 10 days), imipramine group (treated with 20 mg/kg/day by gavage for 10 days). Treatment was initiated on day 1, tumor growth and body weights of mice were measured on day 0, 2, 4, 6, 8, 10 after treatment. Mice were sacrificed on day 10 and tumor tissues were isolated for IHC staining. All experiments were repeated at least three times and complied with the guidance of institutional animal care (IACUC number approval by China Medical University: CMUIACUC-2018-323).

### Immunohistochemistry Staining

Mice were sacrificed on day 10, tumors and livers were fixed with 4% PFA at 4°C, respectively. Paraffin-embedded tumor tissue sections were prepared as 5 μm thickness slices by Bio-Check Laboratories Ltd. (New Taipei City, Taiwan) (20). According to the instructions provided with the EMD Millipore’s IHC Select^®^ kit, the sections were stained with EGFR (Try1068), PKC-δ (Thr507) and NF-κB (Ser536), cleaved caspase-3, -8, and -9, MMP-9, XIAP, and MCL-1 antibodies, respectively. The stained sections were scanned at 200× magnification by using the microscopy-based TissueFAXS platform (TissueGnostics, Vienna, Austria). Positive expression of IHC indices in tumor tissues was quantified with ImageJ software version 1.50 (National Institutes of Health, Bethesda, MD, USA) (21).

### Hematoxylin and Eosin Staining

Mice were sacrificed on day 10 and livers were fixed with 4% PFA at 4°C. Heart, liver, kidney, spleen, lung and intestine tissue for mice were extracted to perform H&E staining (20). Paraffin-embedded tumor tissue section with 5 μm thickness and H&E staining was performed by Bio-Check Laboratories Ltd. (New Taipei City, Taiwan).

### Bioluminescence Imaging

*In vivo*, BLI was conducted on a cryogenically cooled IVIS Lumina LT Series using Living Imaging (Caliper Lifesciences). Mice have received an intraperitoneal injection of 150 mg/kg of luciferin potassium salt dissolved in 100 μl PBS (Gold Biotechnology, St. Louis, MO) and subsequently anesthetized with 5% isoflurane (Abbott, North Chicago, IL). The animals were then placed into the BLI chamber and the anesthesia was continually provided with 2% isoflurane by the nose cone. Images were acquired 15 min after luciferin administration. An integration time of 1 min with a binning of 100 pixels was used for luminescence image acquisition. Signal intensity was quantified as total photon flux (p/s/cm^2^/sr) within the region of interest after subtraction of the background luminescence (22).

### Statistical Analysis

One-way ANOVA was performed in this study to compare the difference between control, erlotinib and imipramine treatment groups by Microsoft excel 2017. The *P*-value that smaller than 0.05 was defined as a significant difference. Each value in this study was displayed as mean ± standard error. Statistical differences between groups were mentioned in each figure legends.

## Results

### Imipramine Significantly Induced Cytotoxicity and Apoptosis of NSCLC Cells

**Figure 1:**
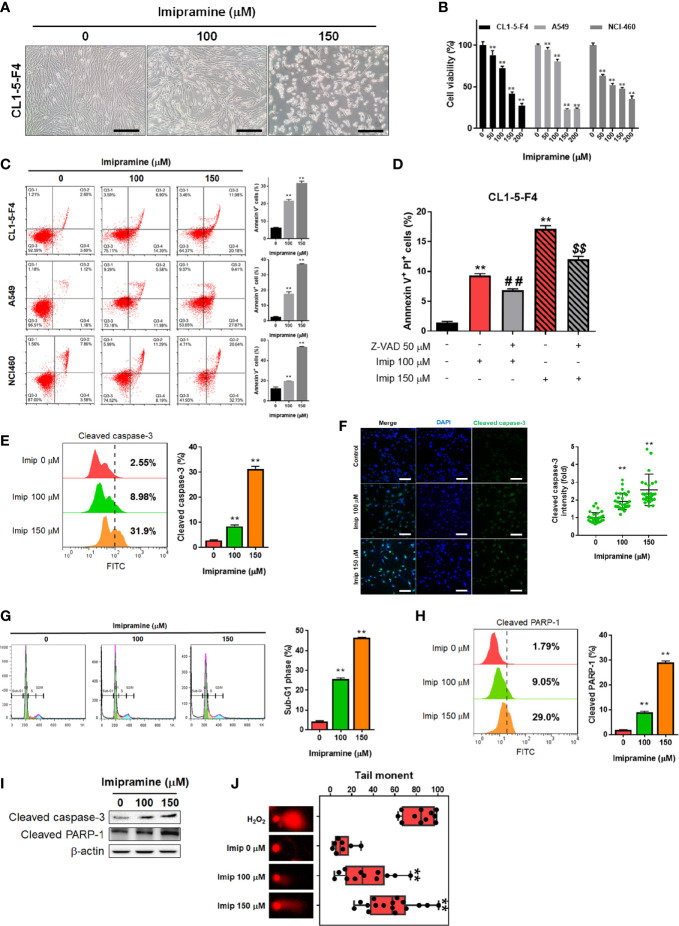
The induction of apoptosis and cytotoxicity of imipramine in NSCLC cells. **(A)** The morphology of CL1-5-F4 cells after 100 and 150 μM imipramine treatment for 48 hr is displayed. **(B)** Cell viability of CL1-5-F4, A549, and NCI460 cells after 0-200 μM imipramine treatment for 48 hr are displayed. **(C)** The Annexin-V/PI staining pattern and quantification results of CL1-5-F4, A549, and NCI460 cells after 100 and 150 μM imipramine treatment for 48 hr are displayed. **(D)** Quantification results Annexin-V positive percentage after Z-VAD combined with 100 and 150 μM imipramine in CL1-5-F4 cells are showed. **(E, G, H)** Staining pattern and quantification results of cleaved caspase-3, cell cycle and cleaved PARP-1 in CL1-5-F4 cells after 100 and 150 μM imipramine treatment for 48 hr are presented. **(F)** IF staining images and quantification results after 100 and 150 μM imipramine treatment in CL1-5-F4 cells are displayed. **(I)** The protein expression of cleaved caspase-3 and cleaved-PARP-1 are displayed. **(J)** One represented comet tail movement from each treatment group is displayed. H_2_O_2_ is represented as the positive control. (^**^ *p* < 0.01 *vs*. 0 μM imipramine; ^##^ and ^$$^ *p* < 0.01 *vs*. imipramine alone).

To evaluate the cytotoxicity of imipramine on NSCLC, we used three NSCLC cell lines (CL1-5-F4, A549, and NCI460) and treated them with 0-200 μM imipramine for 48 hr. CL1-5-F4 cells were showed the cell shrinkage morphology after imipramine treatment (**Figure 1A**). Imipramine also decreased cells viability of three NSCLC cell lines a dose-dependent manner (**Figure 1B**). The Annexin-V^+^ cells were all increased along with dosage rising after imipramine treatment in three NSCLC cells (**Figure 1C**). To further investigated whether imipramine-induced apoptosis was associated with caspase-dependent signaling activation, CL1-5-F4 cells were co-treated with imipramine with Z-VAD, a caspase family inhibitor, and tested by Annexin-V/PI staining. As indicated in late apoptosis population, apoptosis induced by imipramine was markedly reversed by Z-VAD (**Figure 1D**). The activation of cleaved caspase-3 was validated by flow cytometry and IF staining. Results in **Figures 1E, F** indicated that imipramine may effectively induce the activation of cleaved caspase-3. The sub-G1 phase accumulation that represents as apoptotic cells was also found in imipramine-treated CL1-5-F4 cells (**Figure 1G**). Moreover, cleaved PARP-1, an apoptosis marker, were also significantly increased by imipramine (**Figure 1H**). The protein expression of cleaved caspase-3 and cleaved PARP-1 were all increased by imipramine as indicated in **Figure 1I**. Comet assay which revealed DNA damage pattern was effectively increased in imipramine treated CL1-5-F4 cells (**Figure 1J**). All above, we confirmed that imipramine may induce cytotoxicity and apoptosis of NSCLC cells.

### Imipramine Triggered Extrinsic and Intrinsic Apoptosis Signaling in CL1-5-F4 Cells

**Figure 2:**
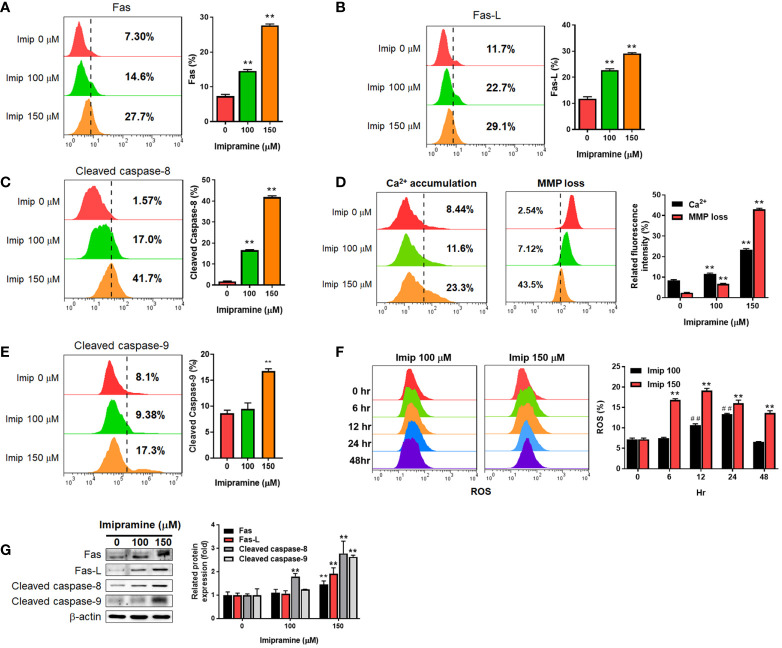
The induction of extrinsic and intrinsic apoptosis by imipramine in CL1-5-F4 cells. **(A–E)** Staining pattern and quantification results of Fas, Fas-L, cleaved caspase-8, Ca^2+^, ΔΨm and cleaved caspase-9 after 100 and 150 μM imipramine treatments for 48 hr are displayed. **(F)** The ROS staining and quantification results of 100 and 150 μM imipramine treatment for 0, 6, 12, 24 and 48 hr are displayed. **(G)** The protein expression pattern and quantification results of Fas, Fas-L, cleaved caspase-8 and cleaved caspase-9 after 100 and 150 μM imipramine treatments for 48 hr are displayed. (^**^ *p* < 0.01 *vs*. 0 μM imipramine; ^##^ *p* < 0.01 *vs*. 0 hr).

To further investigated if both extrinsic and intrinsic apoptosis pathways were affected by imipramine, flow cytometry and Western blotting assays were utilized to test the activation of the following related markers. In the extrinsic apoptosis pathway, Fas, Fas ligand (Fas-L) and cleaved caspase-8 were all increased by imipramine-treated CL1-5-F4 cells (**Figures 2A–C**). Intrinsic apoptotic pathway markers, including accumulation of cellular calcium (Ca^2+^), loss of mitochondria membrane potential (MMP, ΔΨm), cleaved caspase-9 and production of reactive oxygen species (20) were all investigated induced by imipramine (**Figures 2D–F**). Nevertheless, the highest induction percentage of ROS was found after 12-24 hrs treatment of imipramine but not 48 hrs treatment. Additionally, the protein expression of Fas, Fas-L, cleaved caspase-8 and cleaved caspase-9 were also increased by imipramine (**Figure 2G**). Taken together, we indicated that imipramine markedly triggered both extrinsic and intrinsic apoptosis pathways in CL-1-5-F4 cells.

### Imipramine Inhibited NF-κB Activation *via* EGFR/PKC-δ/NF-κB Pathway in CL1-5-F4 Cells

**Figure 3:**
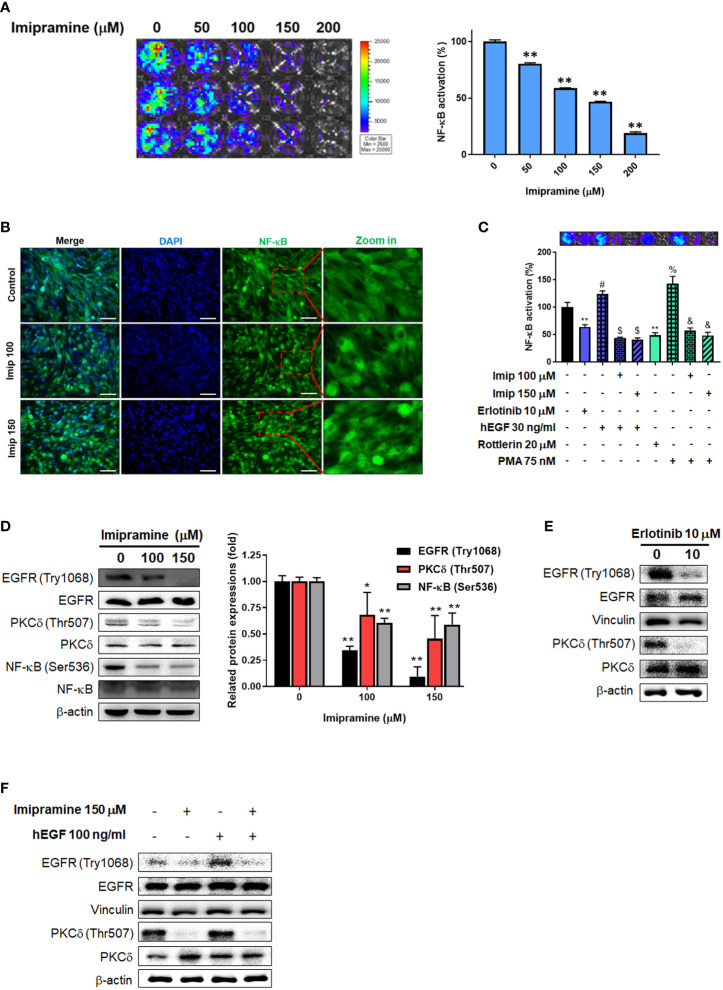
The inhibition of EGFR/PKC-δ/NF-κB pathway by imipramine in CL1-5-F4 cells. **(A)** The NF-κB activation pattern and quantification results of CL1-5-F4/*NF-κB-luc2* cells treated with 0-200 μM imipramine for 48 hr. **(B)** The nuclear translocation results of CL1-5-F4 cells treated with 100 and 150 μM imipramine for 48 hr by IF staining. **(C)** The NF-κB activation pattern and quantification results of CL1-5-F4/*NF-κB-luc2* cells treated with 10 μM erlotinib, 20 nM rottlerin, 100 μM imipramine, 150 μM imipramine alone or combined with 30 ng/ml hEGF and 75 nM PMA. **(D)** The protein expression of EGFR, PKC-δ and NF-κB after 100 and 150 μM imipramine treatment are displayed. The protein expression of EGFR and PKC-δ are treated with **(E)** 10 μM erlotinib or **(F)** 100 ng/ml hEGF, 150 μM imipramine or combined of both are displayed. (^**^ *p *< 0.01 *vs*. 0 μM imipramine).

To investigate the activation of NF-κB after imipramine treatment in CL1-5-F4 cells, we transfected CL1-5-F4 cells with pNF-κB-luciferase reporter gene (pNF-κB-luc2 vector) and performed nuclear translocation assay of NF-κB. Luminescence images indicated the obvious NF-κB activation was suppressed approximately 50-60% by 100-150 μM of imipramine (**Figure 3A**). In addition, we found that the nuclear translocation of NF-κB was reduced by imipramine treatment (**Figure 3B**). In **Figure 3B**, fewer translocated cells were found in 150 μM as compared to 100 μM of imipramine. Then, we try to identify the upstream regulator of NF-κB, we performed NF-κB reporter gene assay after treated with hEGF and PKC-δ inducers and inhibitors in CL1-5-F4/*NF-κB-luc2* cells. Both EGFR inhibitor (erlotinib) and PKC-δ inhibitor (rottlerin) may suppress NF-κB activation (**Figure 3C**). NF-κB which activated by EGFR (hEGF) and PKC inducer (PMA) were be diminished when combining with imipramine (100 and 150 μM). We also identified other upstream regulator of NF-κB including ERK (PD98059) and AKT (LY294002) on NSCLC; however, PKC-δ inhibitor (rottlerin) showed greater NF-κB suppression capacity (**Supplementary Figure**). Furthermore, the protein expressions of EGFR (Try1068), PKC-δ (Thr507), and NF-κB (Ser536) were all downregulated after imipramine treatment (**Figure 3D**). In **Figure 3E**, erlotinib may suppress phosphorylation of EGFR and PKC-δ, which imply the inactivation of NF-κB is associated with the suppression EGFR/PKC-δ. Additionally, imipramine may successfully suppress hEGF-induced EGFR/PKC-δ (**Figure 3F**). According to the above results, we suggested that imipramine suppressed NF-κB activation of CL1-5-F4 cells was associated with the inhibition of the EGFR/PKC-δ pathway.

### Imipramine Suppressed NF-κB Mediated Metastasis Capacity in CL1-5-F4 Cells

**Figure 4:**
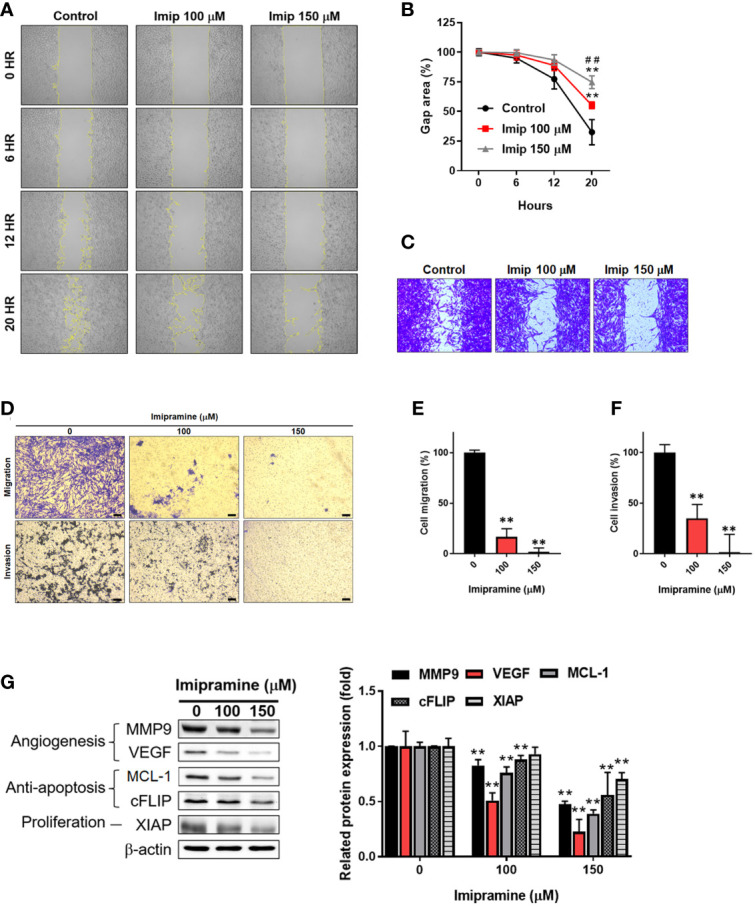
The suppression of NF-κB mediated metastasis ability by imipramine in CL1-5-F4 cells. **(A, B)** The migration pattern and quantification of gap area after treatment with 100 and 150 μM imipramine are performed by wound healing assay and displayed. **(C)** The crystal violet staining results of wound healing assay after 20 hr migration are presented. **(D–F)** The transwell migration, invasion and quantification bar chart after imipramine treatment are displayed. **(G)** The protein expression of MMP9, VEGF, MCL-1, cFLIP, XIAP and their quantification results are displayed. (^**^ *p* < 0.01 vs. 0 μM imipramine; ^##^ *p* < 0.01 *vs*. 100 μM imipramine).

The activation of NF-κB was also recognized as a crucial factor in metastasis (23); thus, we investigated the migration and invasion characteristic of CL1-5-F4 cells by wound healing assay and transwell invasion assay. In 20 hours’ endpoint of wound healing assay, non-treated CL1-5-F4 cells only remained 30% of the gap area, while 150 μM imipramine remain 75% of the gap area (**Figures 4A–C**). In migration assay, the number of migrated CL1-5-F4 cells was decreased by imipramine treatment after 48 hr treatment (**Figures 4D, E**). In the invasion assay, the number of invaded CL1-5-F4 cells was also suppressed by imipramine (**Figures 4D, F**). In addition, we used Western blotting to investigate NF-κB related downstream proteins expression, including VEGF, MMP-9, cFLIP, MCL-1, and XIAP (17). Angiogenesis [vascular endothelial growth factor (VEGF), and metastasis-associated proteins (matrix metalloproteinase-9 (MMP-9)], anti-apoptotic protein [(Cellular FLICE (FADD-like IL-1β-converting enzyme)-inhibitory protein (cFLIP), myeloid cell leukemia-1 (MCL-1)], and proliferation protein [X-linked inhibitor of apoptosis protein (XAIP)], were all reduced by imipramine as showed in **Figure 4G**. In sum, our result suggested that NF-κB mediated metastasis ability and tumor progression associated proteins expression was dose-dependently reduced by imipramine.

### Imipramine Can Inhibit Tumor Growth and NF-κB Activation Without Triggering General Toxicity in CL1-5-F4/*NF-κB-luc2* Cells Bearing Mice

**Figure 5:**
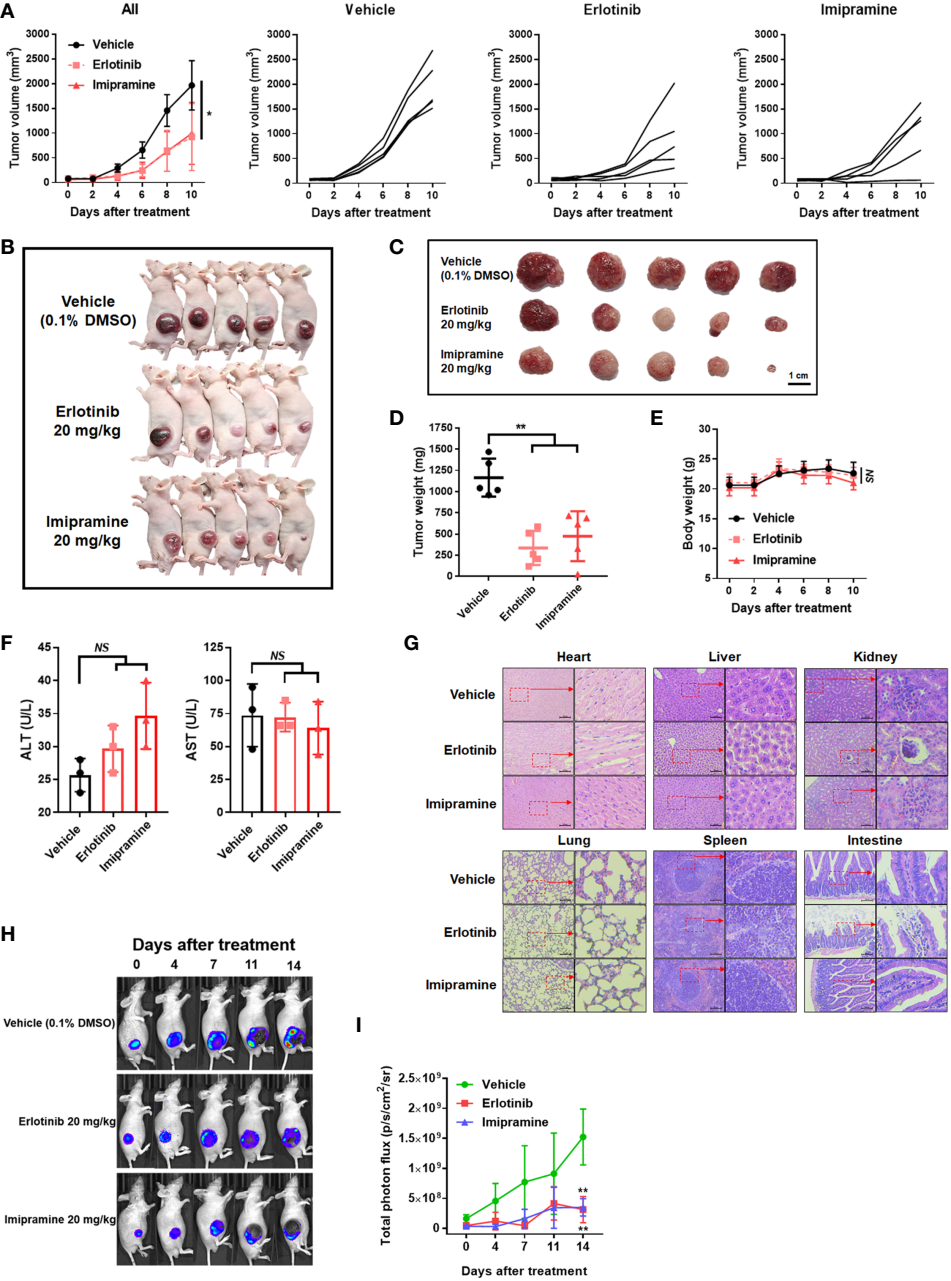
The suppression of tumor growth and NF-κB activation by imipramine in CL-1-5/*NF-κB-luc2* cells bearing mice. **(A)** Average tumor volume and independent tumor volume from each mouse from day 0 to day 10 are displayed. **(B, C)** Photographed of each mouse and extracted tumor on day 10 is presented. **(D)** Mice tumor weight from extracted tumor is presented. **(E)** Mice body weight, **(F)** AST/ALT level form serum, and **(G)** heart, liver, kidney, lung, spleen, intestine pathology on day 10 after imipramine and erlotinib treatment are presented. **(H, I)** NF-κB activation and its quantification results in each group on day 0, 7, 11, 14 are displayed. (^**^ *p* < 0.01 *vs*. vehicle; NS, no significant differences; scale bar =100 μm).

In an animal experiment, we established subcutaneous CL1-5-F4/*NF-κB-luc2* cells bearing model on BALB/c nude mice to validate the treatment efficacy of imipramine. As illustrated in **Figure 5A**, erlotinib and imipramine groups markedly suppressed tumor growth as compared to control. Tumor growth of each mouse was demonstrated similar growth inhibition pattern after treated with imipramine and erlotinib. The smallest tumor was found in the imipramine treatment group on day 10 as compared to control groups (**Figures 5B, C**). Moreover, the mean tumor growth time of imipramine is 10.8 day, which is markedly smaller than control group (**Table 1**). The mean tumor growth delay time of imipramine treated group is 5.5 day less than control. The mean growth inhibition rate of imipramine treated group is 2.04 times smaller than control group. Imipramine also markedly decreased the weight of tumor (**Figure 5D**). The statistical result of mice body weight showed no significant difference between the 3 groups (**Figure 5E**). The level of AST, ALT and γGT in serum was used to identify the function of liver. As showed in **Figure 5F**, the level of AST and ALT remained unchanged after imipramine treatment as compared to vehicle. The level of γGT which represent as liver function was all smaller than 4 (**Table 2**). However, in heart, liver, kidney, lung, spleen, and intestine pathology results, the chromatin precipitation in liver tissue was only found in the erlotinib treatment group but not in the control group and imipramine group (**Figure 5G**). This result indicated that erlotinib showed noticeable liver toxicity as compared to imipramine. In addition, the NF-κB activation within the tumor was effectively decreased by erlotinib and imipramine (**Figures 5H, I**). In sum, tumor growth and NF-κB activation were both effectively suppressed by imipramine treatment.

**Table 1:** Mean tumor growth time, delay time, and inhibition rate in CL1-5-F4 tumor bearing mice after treatment with erlotinib, and imipramine.

Treatment	MTGT (day)^*^	MTGDT (day)^#^	MGIR^$^
**Vehicle**	5.30	NA	NA
**Erlotinib**	11.88	6.58	2.24
**Imipramine**	10.79	5.49	2.04

NA, not available.

^*^Mean tumor growth time (MTGT): the time at which the tumor volume reached 1000 mm^3^.

^#^Mean tumor growth delay time (MTGDT): the mean tumor growth time of the treated group minus that of the control group.

^$^Mean growth inhibition rate (MGIR): the mean tumor growth time of the treated group/the mean tumor growth time of the sham control group.

**Table 2:** The serum level of gamma-glutamyl transferase (γGT).

Group	γGT (U/L)
**Vehicle-1**	4
**Vehicle-2**	3
**Vehicle-3**	3
**Erlotinib-1**	<3
**Erlotinib-2**	<3
**Erlotinib-3**	3
**Imipramine-1**	<3
**Imipramine -2**	<3
**Imipramine -3**	<3

### Imipramine Inhibited Tumor Growth Was Associated With EGFR/PKC-δ/NF-κB Pathway Inactivation, Extrinsic and Intrinsic Apoptosis Induction

**Figure 6:**
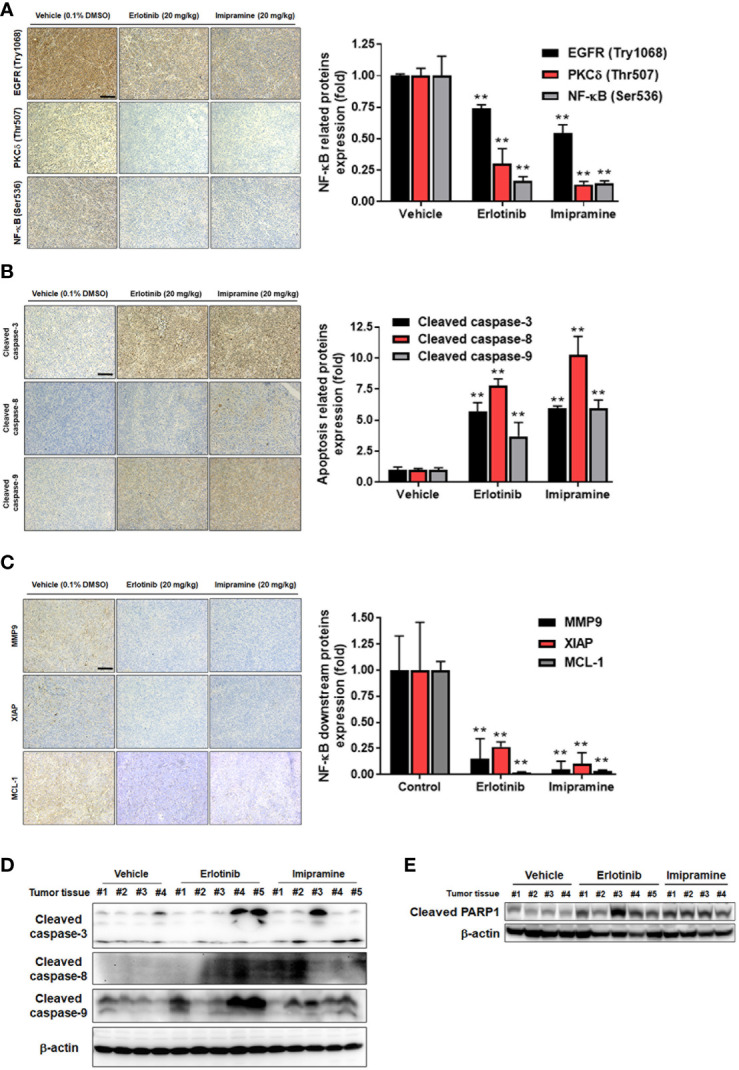
The inhibition of EGFR/PKC-δ/NF-κB proteins phosphorylation and induction of apoptosis-related proteins by imipramine in CL1-5-F4/*NF-κB-luc2* bearing tumor. **(A–C)** The protein expression from IHC of EGFR (Try 1068), PKC-δ (Thr507), NF-κB (Ser536), cleaved caspase-3, -8, -9, MMP-9, XIAP, MCL-1 and their quantification bar chart are presented. **(D, E)** The tumor *ex vivo* Western blotting from each mice of cleaved caspase-3, -8, -9 and PARP-1 is presented. (^**^ *p* < 0.01 *vs*. vehicle; scale bar =100 μm).

Here, we also used IHC staining to observe whether EGFR/PKC-δ/NF-κB signaling was suppressed by imipramine in CL1-5-F4*/NF-κB-luc2* bearing tumor. The phosphorylation of EGFR, PKC-δ, and NF-κB was significantly decreased 50-80% by imipramine (**Figure 6A**). Three apoptotic markers included general apoptosis protein-activated caspase-3, extrinsic apoptosis protein-activated caspase-8, and intrinsic apoptosis protein-activated caspase-9 in the tumor section were all markedly increased by imipramine and erlotinib (**Figure 6B**). Moreover, NF-κB downstream protein expressions such as MMP9, MCL-1 and XIAP were all reduced by imipramine as displayed in **Figure 6C**. Additionally, the protein expression from extracted tumor also demonstrated the induction of cleaved caspase-3, 8, -9 and PARP-1 by imipramine (**Figures 6D, E**). In conclusion, these results revealed that imipramine not only suppressed the phosphorylation of EGFR/PKC-δ/NF-κB signaling and protein expression of NF-κB-mediated downstream proteins but also significantly induced the protein expression of apoptotic related proteins in CL1-5-F4*/NF-κB-luc2* bearing mice tumor.

## Discussion

The effectiveness of imipramine in SCLC cell growth inhibition has been revealed by experiments with cell and animal models. Imipramine inhibits tumor growth through the induction of apoptosis and blockade of survival signaling of SCLC *in vitro* and *in vivo* (13). In this study, we demonstrate the efficacy of imipramine against NSCLC cell growth, survival, and invasion. In addition, we also elucidated a potential anti-NSCLC mechanism of imipramine.

Increased expression of EGFR phosphorylation has been recognized as an unfavorable prognostic predictor associated with poor outcome of NSCLC (24). EGFR inhibitors such as gefitinib, erlotinib, afatinib, and osimertinib are approved for the treatment of EGFR mutation-positive NSCLC patients (25). In addition, erlotinib has also been shown to provide a survival benefit in NSCLC patients with wild-type EGFR (26). Our data demonstrated that imipramine significantly decreased the expression of p-EGFR in NSCLC cells *in vitro* and *ex vivo* (**Figures 3D**, **6A**). EGF-activated EGFR was also attenuated with imipramine treatment (**Figure 3F**). Both imipramine and erlotinib effectively diminished tumor growth in CL-1-5-F4-bearing mice. Notably, the mean tumor size of the experimental group treated with imipramine showed similar tumor inhibition potential with the experimental group treated with the same erlotinib dosage alone (**Figures 5A–D**).

Protein kinase C (PKC)-delta (PKCδ), a member of the protein kinase C family, mediates cell survival, proliferation, and invasion of NSCLC cells. PKCδ inhibition by short hairpin RNA (shRNA) and rottlerin have been indicated to suppress growth and invasion of NSCLC cells and sensitize NSCLC cells to chemotherapy, respectively (27, 28). NF-κB, a heterodimeric transcription factor composed of p50 and p65 subunits, on the other hand, can be activated by upstream kinases to upregulate the expression of oncogenes (29). Previous studies presented that QNZ, a NF-κB inhibitor, reduced NSCLC cell invasion and the protein expression of MMP-9, VEGF, MCL-1, C-FLIP and XIAP through NF-κB inactivation (17, 30). Corresponding to our results, imipramine effectively inhibited PKCδ and NF-κB activation as well as cell migration and invasion (**Figures 3D**, **4A–F**, **6C**). Expression of the above-mentioned invasion-associated and anti-apoptotic proteins was also significantly decreased by imipramine treatment (**Figure 4G**).

Both EGFR and PKC-δ signaling interplay with NF-κB-mediated tumor progression (31–33). Nuclear localization of PKC-δ is required for EGFR activation-induced NF-κB phosphorylation in NSCLC cells (31). These findings prompted us to verify the relationships among the EGFR (Try1068), PKC-δ (Thr507), and NF-κB (Ser536) signaling. We found that hEGF significantly upregulated the expression of EGFR (Try1068), PKC-δ (Thr507), and NF-κB (Ser536) signaling in CL-1-5-F4 cells while the protein level of PKC-δ (Thr507) was decreased by erlotinib treatment (**Figure 3E, F**). In addition, NF-κB signaling was upregulated and downregulated by PMA and rottlerin treatment, respectively. These findings suggested that PKC-δ (Thr507) participates in EGFR (Try1068)-potentiated NF-κB signaling. In addition to suppression of endogenous EGFR signaling, EGF-induced EGFR/PKC-δ/NF-κB signaling transduction was abolished with imipramine treatment (**Figure 3F**).

Overexpression of anti-apoptotic proteins eliminates the anti-cancer efficacy of therapeutic agents through the blockade of extrinsic and intrinsic apoptotic pathways. Initiation of apoptotic pathways and inhibited expression of anti-apoptotic proteins by using complementary agents enhance chemotherapy- and radiotherapy-induced tumor regression (34–36). Afatinib, an EGFR tyrosine kinase inhibitor, has been demonstrated to promote vinorelbine (a chemotherapeutic agent)-induced apoptosis through initiating the intrinsic pathway and inhibiting expressions of anti-apoptotic proteins, such as B-cell lymphoma 2 (Bcl-2) and B-cell lymphoma-extra-large (Bcl-xL), in NSCLC cells (36). In addition to inhibition of anti-apoptotic protein expression, we also found that imipramine effectively triggered apoptosis through the extrinsic and intrinsic pathways (**Figure 2**). As Z-VAD (a caspase-family inhibitor) significantly inhibited imipramine-induced late apoptosis, a caspase-dependent pathway was hence identified to be involved in imipramine-induced apoptosis (**Figure 1D**).

## Conclusion

In conclusion, this study presented that downregulation of the EGFR/PKC-δ/NF-κB signaling is related to the imipramine-inhibited progression of NSCLC. We suggested that imipramine as a potential complementary agent may confer NSCLC patients with therapeutic benefits. Imipramine, as a potential complementary agent, may confer NSCLC patients with therapeutic benefits through induction of apoptosis in addition to its suppression of tumor growth, survival and invasion.

## Data Availability Statement

The original contributions presented in the study are included in the article/**Supplementary Material**. Further inquiries can be directed to the corresponding authors.

## Ethics Statement

The animal study was reviewed and approved by Animal Care and Use Committee at China Medical University.

## Author Contributions

Conceptualization, C-HC, K-LL and F-TH. Data curation, P-FY and F-TH. Funding acquisition, W-TC, C-HC and F-TH. Supervision, C-HC, K-LL, and F-TH. Validation, P-FY, Y-HL, I-TC, and W-TC. Writing – original draft, P-FY, Y-HL, and I-TC. Writing – review and editing, K-LL and F-TH. All authors contributed to the article and approved the submitted version.

## Funding

This study was financially supported by a grant from the Ministry of Science and Technology, Taipei, Taiwan [grant number: MOST 108-2314-B-039-007-MY3], Zuoying Branch of Kaohsiung Armed Forces General Hospital, Kaohsiung, Taiwan [grant number: KAFGH-ZY-A-109020] and Show Chwan Memorial Hospital, Changhua, Taiwan [grant number: SRD-108006], respectively. This work was also financially supported by the “Drug Development Center, China Medical University” from The Featured Areas Research Center Program within the framework of the Higher Education Sprout Project by the Ministry of Education (MOE) in Taiwan.

## Conflict of Interest

The authors declare that the research was conducted in the absence of any commercial or financial relationships that could be construed as a potential conflict of interest.

## Publisher’s Note

All claims expressed in this article are solely those of the authors and do not necessarily represent those of their affiliated organizations, or those of the publisher, the editors and the reviewers. Any product that may be evaluated in this article, or claim that may be made by its manufacturer, is not guaranteed or endorsed by the publisher.

## Supplementary Material

Supplementary Figure The NF-κB activation was performed by NF-κB reporter gene assay. The CL1-5-F4/*NF-κB-luc2* cells were treated with 20 μM LY294002 and PD98059 for 48 hr and assayed by IVIS. The relative NF-κB activation was normalized by cell viability.
