# Supplementary material for: Suppression of EGFR/PKC-δ/NF-κB Signaling Associated With Imipramine-Inhibited Progression of Non-Small Cell Lung Cancer
Source: Front Oncol. 2021 Oct 26;11:735183. doi: 10.3389/fonc.2021.735183 (PMC8576332; doi:10.3389/fonc.2021.735183)

**Suppression of EGFR/PKC-δ/NF-κB signaling associated with imipramine-inhibited progression of non-small cell lung cancer**

**Supplementary figure**

**

**

Western blotting full blot images


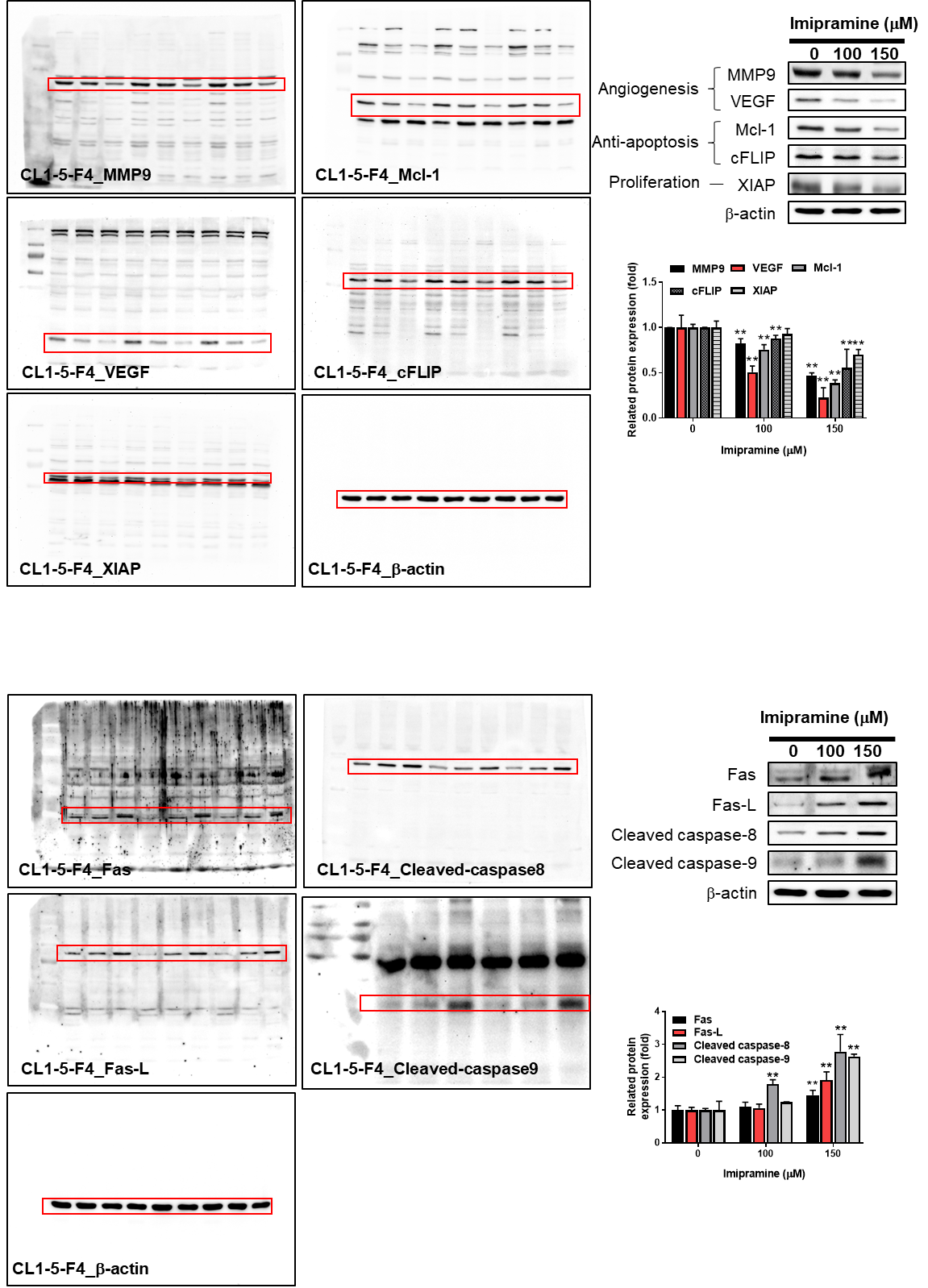


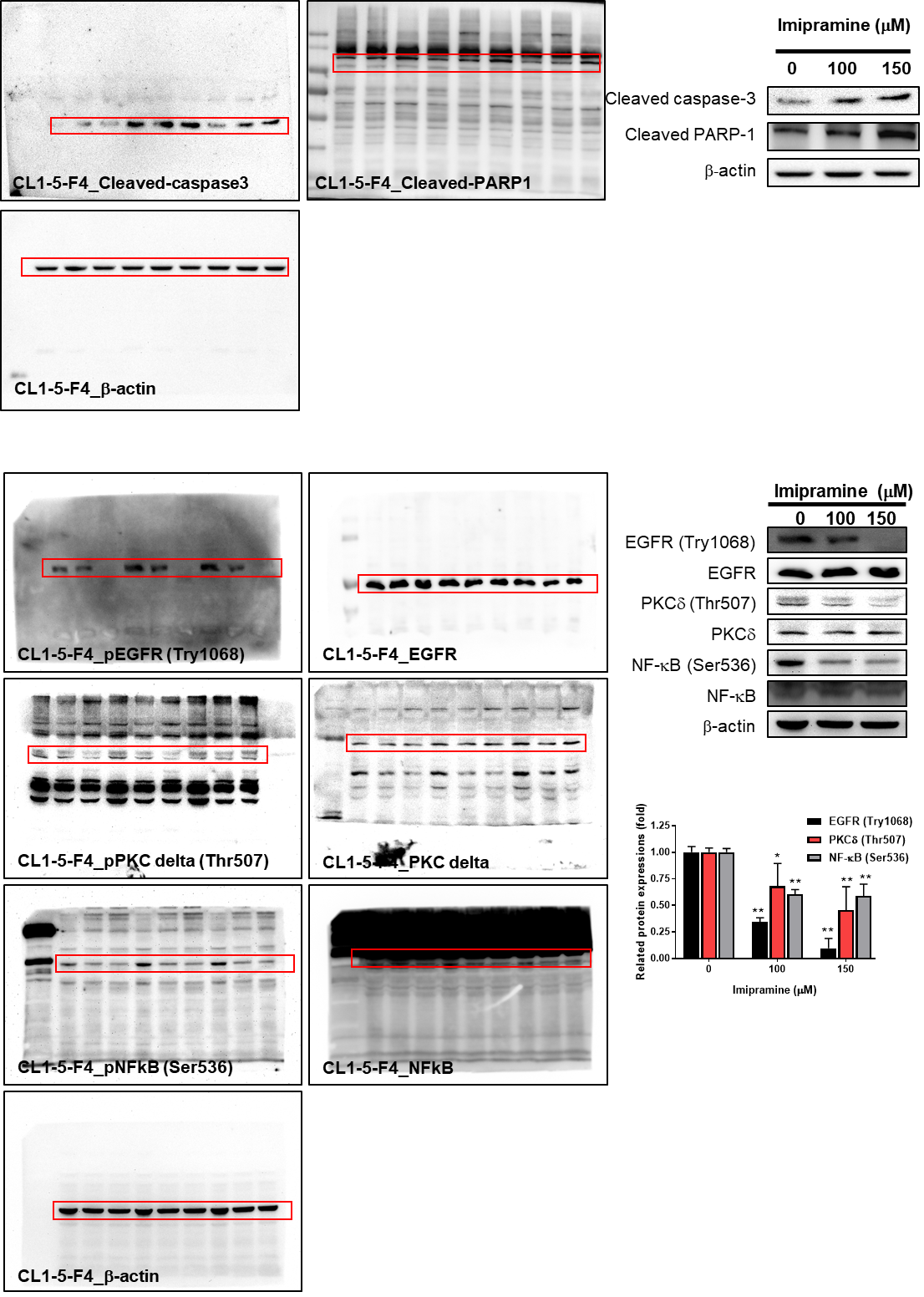


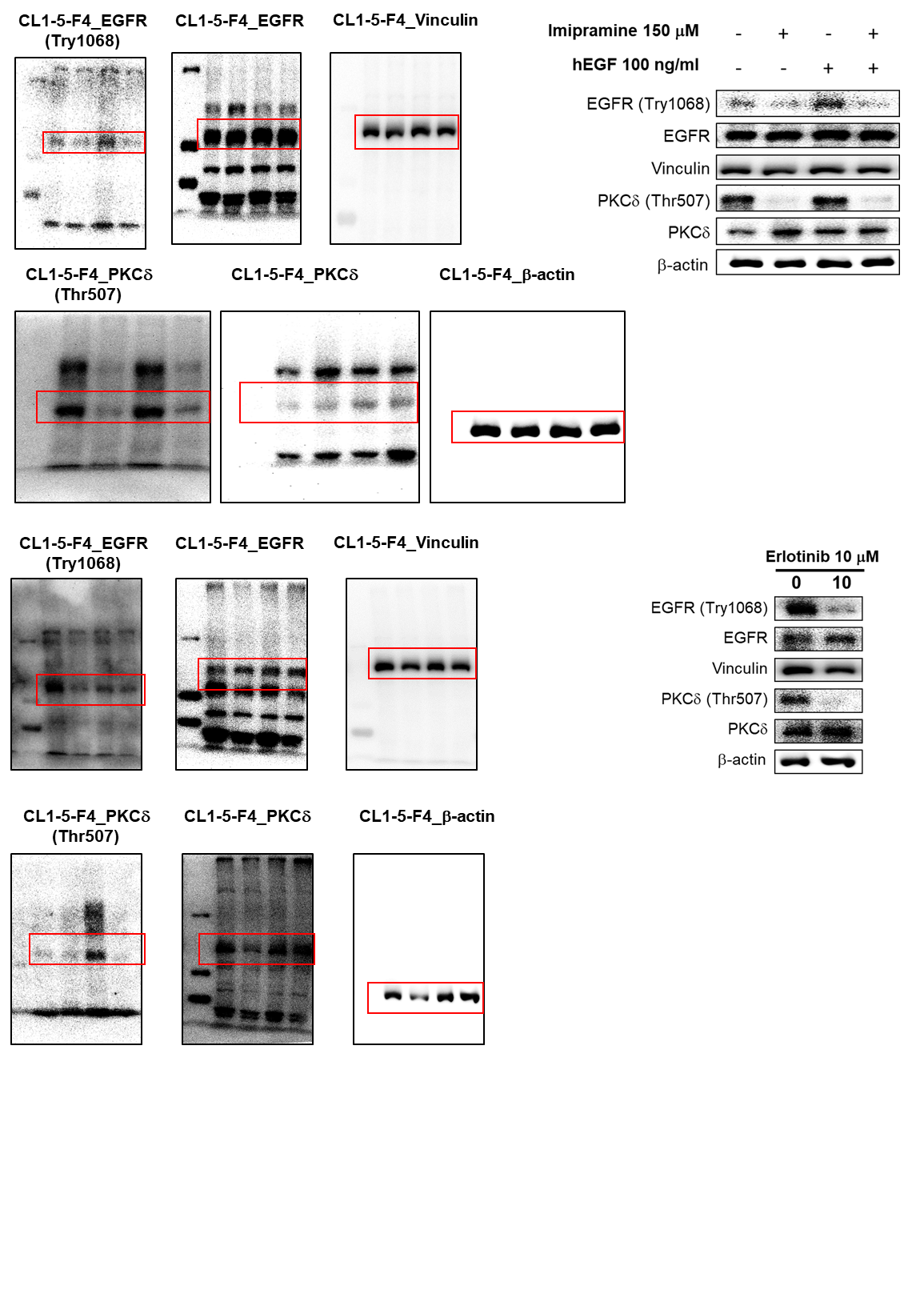


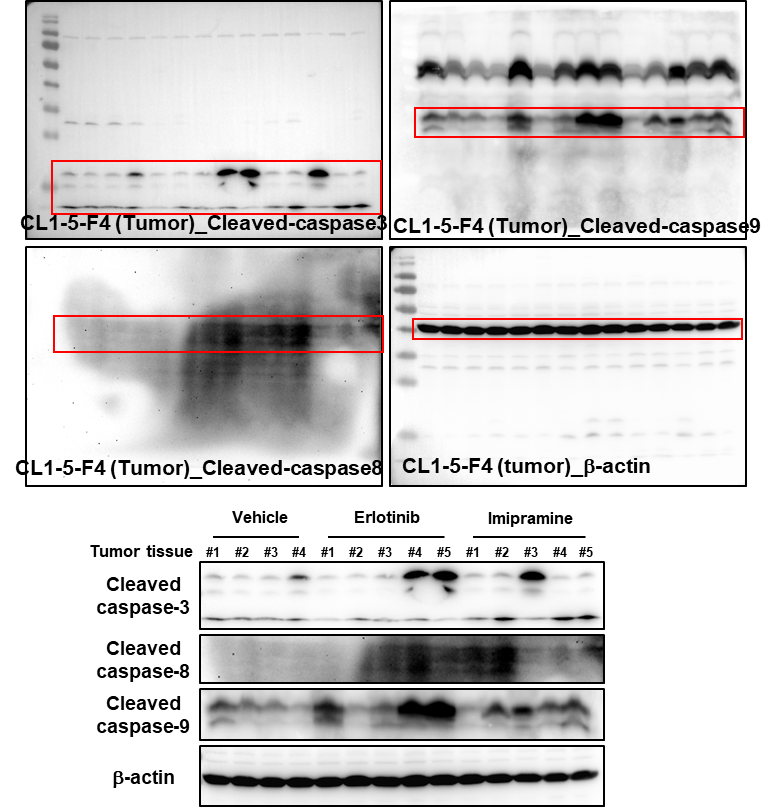


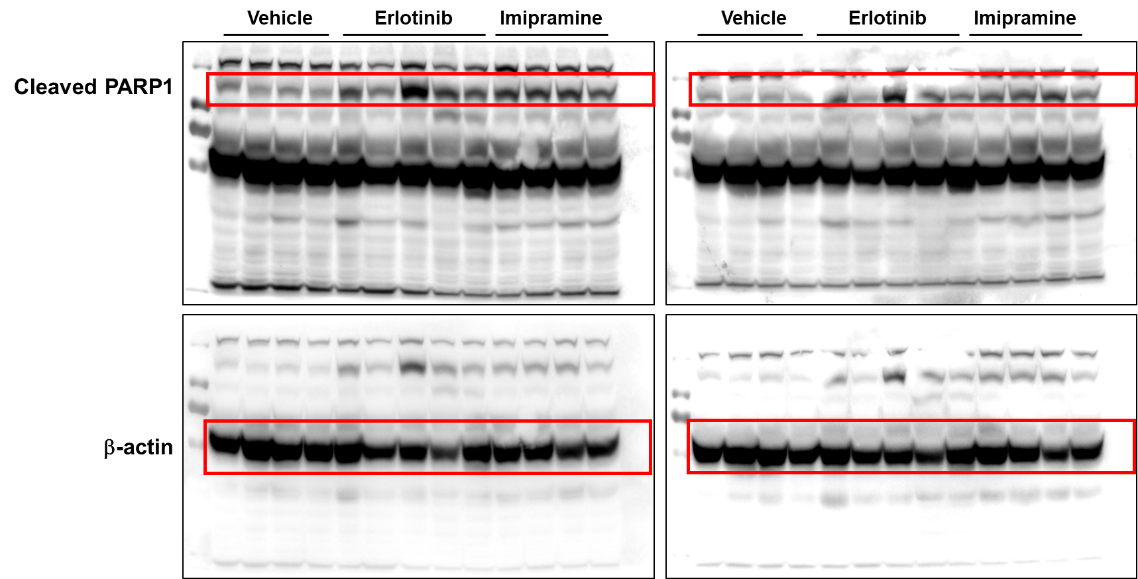


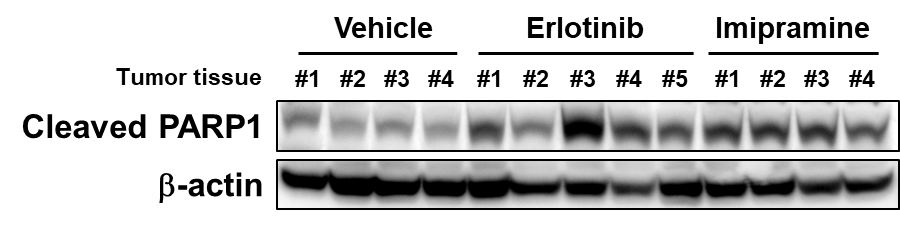

Supplement: Supplementary Figure — The NF-κB activation was performed by NF-κB reporter gene assay. The CL1-5-F4/NF-κB-luc2 cells were treated with 20 μM LY294002 and PD98059 for 48 hr and assayed by IVIS. The relative NF-κB activation was normalized by cell viability. [file DataSheet_1.docx]
